# Alcohol Dependence Associated with Increased Utilitarian Moral Judgment: A Case Control Study

**DOI:** 10.1371/journal.pone.0039882

**Published:** 2012-06-28

**Authors:** Lotfi Khemiri, Joar Guterstam, Johan Franck, Nitya Jayaram-Lindström

**Affiliations:** Division of Psychiatry, Department of Clinical Neuroscience, Karolinska Institutet, Stockholm, Sweden; University of Granada, Spain

## Abstract

Recent studies indicate that emotional processes, mediated by the ventromedial prefrontal cortex (VMPC), are of great importance for moral judgment. Neurological patients with VMPC dysfunction have been shown to generate increased utilitarian moral judgments, i.e. are more likely to endorse emotionally aversive actions in order to maximize aggregate welfare, when faced with emotionally salient personal moral dilemmas. Patients with alcohol dependence (AD) also exhibit impairments in functions mediated by the prefrontal cortex, but whether they exhibit increased utilitarian moral reasoning has not previously been investigated. The aim of this study was to investigate moral judgment in AD patients (n = 20) compared to healthy controls (n = 20) matched by sex, age and education years. Each subject responded to a battery of 50 hypothetical dilemmas categorized as non-moral, moral impersonal and moral personal. They also responded to a questionnaire evaluating explicit knowledge of social and moral norms. Results confirmed our hypothesis that AD patients generated increased utilitarian moral judgment compared to controls when faced with moral personal dilemmas. Crucially, there was no difference in their responses to non-moral or impersonal moral dilemmas, nor knowledge of explicit social and moral norms. One possible explanation is that damage to the VMPC, caused by long term repeated exposure to alcohol results in emotional dysfunction, predisposing to utilitarian moral judgment. This work elucidates a novel aspect of the neuropsychological profile of AD patients, namely a tendency to generate utilitarian moral judgment when faced with emotionally salient moral personal dilemmas.

## Introduction

Charles Darwin wrote, “Of all the differences between man and the lower animals, the moral sense or conscience is by far the most important” [1]. Even though there is an ongoing discussion regarding how fundamental this difference actually is [2], it is obvious that human beings have a capacity for moral reasoning (i.e. reasoning concerning the properties of right, wrong, permissibility, blame etc.) that goes beyond that of any other social animal. Human morality has traditionally been a topic for moral philosophers, but recently cognitive neuroscientists have started to empirically investigate the psychological and neurobiological processes underlying our moral judgments. In contrast to a traditional rationalistic view of moral reasoning as a product of conscious reasoning [3], [4], these studies indicate that emotional processes mediated by the ventromedial prefrontal cortex (VMPC), are of great importance in moral judgment [5]–[12].

An important inspiration for the entire field of empirical moral psychology is a group of well known philosophical dilemmas known as the “trolley problems” [13], [14]. In the “switch” dilemma, a runaway trolley is heading towards five people laying on the railroad tracks. The only way to stop the trolley from killing these five people is to hit a switch, and thereby diverting the train onto a side track where one person is laying. Thus, in doing so the train will run over and kill one person instead of five. A contrasting dilemma is the “footbridge” dilemma in which a runaway trolley once again threatens to kill five people laying on the railroad tracks. In this dilemma however, the only way to save five lives is to sacrifice one person by pushing him off a footbridge down on the tracks, thus stopping the runaway trolley. Greene and colleagues [7] used functional magnetic resonance imaging (fMRI) to investigate the role of emotion in moral judgment, and why people in general find it morally acceptable to sacrifice one person to save five persons in the “switch” but not in the “footbridge” dilemma. In order to investigate this question they created two sets of moral dilemmas entitled impersonal and personal, containing the relevant features of the “switch” and “footbridge” dilemmas respectively. Personal dilemmas, analogous to the “footbridge” dilemma, involve actions which cause serious bodily harm to a particular person (or group), and the harm is not a result of avoiding an existing threat. The other dilemmas involving actions lacking these features were classified as impersonal [15]. However, it should be noted that the nature of the distinction between personal and impersonal dilemma has been questioned (for discussion, see [15], [16]).

Greene and colleagues [7] were the first to use fMRI to investigate the underlying neural activity in healthy subjects when faced with different kinds of moral dilemmas. The results showed that contemplation of moral impersonal dilemmas led to increased neural activity in brain areas associated with cognition: the dorsolateral prefrontal cortex and inferior parietal lobe. In contrast, contemplation of moral personal dilemmas produced increased neural activity in three brain areas associated with emotion: the posterior cingulate cortex, amygdala and the VMPC [7], [8]. However, when faced with certain kinds or moral dilemmas these two processes are in conflict with each other, as in the “Plane Crash” dilemma:

*Your plane has crashed in the Himalayas. The only survivors are yourself, another man, and a young boy. The three of you travel for days, battling extreme cold and wind. Your only chance at survival is to find your way to a small village on the other side of the mountain, several days away.*

*The boy has a broken leg and cannot move very quickly. His chances of surviving the journey are essentially zero. Without food, you and the other man will probably die as well. The other man suggests that you sacrifice the boy and eat his remains over the next few days.*

*Would you kill this boy so that you and the other man may survive your journey to safety?*

On the one hand the boy will die no matter what, so it is better that two people survive instead of none (cognitive response). On the other hand, most people experience a strong emotional aversion towards killing and eating an innocent person (emotional response). Greene and colleagues have proposed a ‘dual-process theory of moral judgment’, stating that these two separate psychological processes, cognition and emotion, determine the outcome of our moral judgment [8]. In order to generate a utilitarian moral judgment, the initial negative emotional response has to be overridden by cognitive processes. This conflict reflects not only two internal competing processes of cognition and emotion, but also two major views in moral philosophy namely utilitarianism and deontology respectively. Utilitarianism is the normative consequentialist moral theory first formally stated in the eighteenth century by the English philosopher Jeremy Bentham [17]. The moral creed of utilitarianism is often described as follows: One should act so as to maximise the sum of total welfare of everyone affected by one's actions. Deontology in contrast is concerned with concepts such as absolute rights and states that certain actions, e.g. killing, are inherently wrong and may never be performed no matter the consequences [3]. Empirical studies have showed that cognitive processes drive utilitarian moral judgments, while deontological moral judgments on the other hand are fuelled by emotional reactions [7], [8], [11], [18], [19].

Studies of neurological clinical populations with VMPC dysfunction have confirmed the important role of VMPC in moral judgment. Patients with frontotemporal dementia [20] and patients with selective VMPC lesions [18], [19] are more likely to generate utilitarian moral judgments when faced with emotionally salient moral dilemmas. Thus, neurological clinical populations with impaired VMPC function have been shown to generate increased utilitarian moral judgment. The aim of the present study was to investigate moral judgment in a *psychiatric* clinical population with impaired prefrontal function, namely patients with alcohol dependence (AD).

AD is a chronically relapsing disorder characterized by physiological dependence, compulsion to seek and drink alcohol as well as loss of behavioural control, manifested as continued intake of alcohol despite negative consequences [21]. The prefrontal cortex (PFC), which is essential for behavioural control (e.g. planning, motivation, attention and inhibition of impulsive response), is functionally impaired in patents with AD [22], [23].

There are several proposed neuropsychological models explaining the neuropsychological profile of AD patients. The ‘frontal lobe hypothesis’, postulating that the PFC is specifically vulnerable to the neurotoxic effects of alcohol, has received strong empirical support from anatomical, clinical and neuroimaging studies [24], [25]. AD patients exhibit impairments in functions associated with activation of the PFC e.g. increased impulsivity and risky decision making [26], [27], impaired emotional facial perception [28]–[30], emotional prosody perception [31] and humour processing [32], [33]. There is also growing evidence for similarities in decision making between substance abusers and VMPC lesion patients, indicating that these patients share an underlying emotional dysfunction (for review, see [34]). For instance, a majority of AD patients exhibit similar behavioural and physiological response as VMPC lesion patients in the Iowa Gambling Task [35]–[37]. The frontal lobe hypothesis is also supported by neuroanatomical and imaging studies, indicating that chronic AD patients have widespread structural brain changes [38]. Volume loss of grey and white matter was observed in several parts of the brain of AD patients, but the frontal lobes seem to be particularly affected [38]–[40].

In this study we investigated moral judgment in AD patients compared to matched healthy controls. Since previous research has indicated that AD patients have impaired PFC function [24], [25], and also exhibit deficits in decision making similar to VMPC lesion patients [34], we hypothesized that AD patients would generate increased utilitarian moral judgment, similar to patients with VMPC lesions [18], [19]. Specifically, the hypothesis was that AD patients would be more likely to generate utilitarian moral judgments when faced with emotionally salient personal moral dilemmas, and that this difference would be greatest for the “high-conflict” subgroup of personal dilemmas. We further hypothesized that AD patients would have intact knowledge of explicit social and moral norms, and that there would be no difference in the responses to non-moral and impersonal moral dilemmas.

## Methods

### Participants

A naturalistic sample of AD patients (n = 20; all male) was recruited from two addiction rehabilitation centres in Stockholm, Sweden. Inclusion criteria were: 1) Age between 35–70 years; 2) Fulfils at least 5 DSM-IV criteria for AD; 3) Alcohol free since at least 14 days; 4) History of at least 5 years of AD; 5) Willing to give informed consent to participate in the study. Exclusion criteria were: 1) Fulfils DSM-IV criteria for any other substance dependence disorder (except nicotine); 2) Fulfils DSM-IV criteria for any other major psychiatric illness e.g. major depression, schizophrenia or bipolar disorder; 3) History of severe head trauma or stroke; 4) Presence of any neurological disorder e.g. Wernicke-Korsakoff; 5) Traces of alcohol or any other psychoactive substance (central stimulant amines, THC, benzodiazepines, opioids, cocaine) on the day of the testing, confirmed by breathalyser or urine dip test.

Healthy controls (n = 20; all male) matched by sex, age and education years were recruited through local community advertisements and by word of mouth. All healthy controls went through the same study procedure as the AD patients, and they also performed the performed the widely used Alcohol Use Disorder Identification Test (AUDIT) and the corresponding test for narcotic drugs (DUDIT) to exclude alcohol and drug abuse. One healthy subject was excluded from the study because of responding wrongly to one of the non-moral dilemmas used as control questions (see below for further details).

### Materials and Procedure

The study protocol was approved by the Regional Ethics Review Board in Stockholm and all participants provided written informed consent. All subjects were interviewed using the MINI- International Neuropsychiatric Interview [33] to confirm AD and exclude any other major psychiatric pathology. The subjects also completed a questionnaire to screen for any exclusion criteria, a socio-demographic questionnaire, the Montgomery-Asberg Depression Rating Scale (MADRS) self-assessment of depressive symptoms [34] and the Lifetime Drinking History (LDH) interview [35]. Sobriety was confirmed using alcohol breathalyzer and urine dip test was used to detect any illicit psychoactive substance.

Knowledge of explicit social and moral norms was evaluated using 15 items (see Table 1) selected from the Moral Behaviour Inventory used by Mendez et al. [16], originally from the Moral Behaviour Scale [36]. Each item was presented in paper form as follows: “How wrong is it if you” followed by an action e.g. “Take the last seat on a crowded bus” or “Keep over-change in a store”. Subjects responded to each item by choosing “not wrong”, “mildly wrong”, “moderately wrong” or “severely wrong”, on a 4-point rating scale.

**Table 1:** Knowledge of explicit social and moral norms was evaluated using 15 items from the Moral Behaviour Scale [16], to which the subjects responded by choosing “not wrong”, “mildly wrong”, “moderately wrong” or “severely wrong”, on a 4-point rating scale.

How wrong is it if you…
1) Fail to keep minor promises
2) Take the last seat on a crowded bus
3) Sell someone a defective car
4) Drive after having one drink
5) Cut in line when in a hurry
6) Don't give blood during blood drives
7) Are mean to someone you don't like
8) Say a white lie to get a reduced faire
9) Drive out the homeless from your neighbourhood
10) Not help someone pick up their dropped papers
11) Keep over-change at a store
12) Not offer to help after an accident
13) Ignore a hungry stranger
14) Fail to vote in minor elections
15) Keep money found on the ground

Moral judgment was evaluated using 50 hypothetical dilemmas. The dilemmas were divided into three categories based on their content: non-moral (n = 18), moral impersonal (n = 11) and moral personal dilemmas (n = 21). The non-moral dilemmas pose neutral questions, e.g. whether to cut vegetables or boil water first when preparing dinner. Moral impersonal dilemmas are less emotional (e.g. keeping money found in a lost wallet) compared to moral personal dilemmas, which are putatively more emotional in content (e.g. throwing people off a sinking life boat). The moral personal dilemmas were further subdivided into “low-conflict” (n = 9) and “high-conflict” (n = 12), based on their fast and slow response time in healthy volunteers, respectively [15]. The battery of dilemmas and their classification was directly adapted and translated from Koenigs et al. [15], and the complete battery of dilemmas is available online at: http://www.ncbi.nlm.nih.gov/pmc/articles/PMC2244801/bin/NIHMS38394-supplement-supplement.doc.

Each dilemma was presented to the subject through a series of three screens of text (see the ‘plane crash’ dilemma described above as an example). The first two screens described the scenario, and the last one asked the subject what he would do in this situation. The questions were all in the form of: “Would you… in order to …?”. In the moral dilemmas, the questions were constructed so that ‘yes’ responses meant endorsing the proposed utilitarian action, while ‘no’ indicated a deontological judgment. The subjects worked through the text screens by themselves with the space bar, and responded by pushing a “yes” or “no” button on the computer. The subjects were instructed to read the dilemmas in their own pace, and there was no time limit on reading the text screens describing the scenario or responding. Response time recording started when the final screen was presented, and response times above 35 seconds were excluded (n = 12). Two of the non-moral dilemmas called “Turnips” and “Reversed turnips” were used as control dilemmas, since they both had an obvious correct answer i.e. preferring the greater amount of turnips instead of the smaller by responding ‘yes’ and ‘no’ respectively. All participants responded correctly to both these dilemmas except one healthy control who was therefore excluded from the analysis.

### Statistical Analysis

Characteristics of patients and healthy controls, including Lifetime Drinking History data, MADRS score and average response to the Moral Behaviour Inventory were analysed using independent two-tailed t-tests with α = 0.05. Smoking status between groups was analysed using chi-square analysis with Yates correction. Mixed analysis of variance (ANOVA) with Group (AD, healthy controls) as between-subject factor and Dilemma (moral, impersonal moral, personal moral) and response type (yes, no) as within-subject factors, was used to analyse proportion of ‘yes’ responses as well as response time. Planned comparisons between groups regarding proportion of ‘yes’ responses as well as response time for different types of dilemmas were analysed using t-tests. Probabilities were two-tailed with an α-level of 0.05.

## Results

Participant characteristics are described in Table 2. There was no significant difference between AD patients and healthy controls regarding age (t = 0,768; P = 0.447) or education years (t = −0.974; P = 0.336). The Lifetime Drinking History interview confirmed that the AD patients had significantly more lifetime drinks (t = 7.091; P = 0.000) and lifetime drinking days (t = 6.316; P = 0.000), but there was no statistically significant difference in age of drinking onset (t = −1.557; P = 0.128). Also, AD patients smoked significantly more than the healthy controls (75% vs 21%; P = 0.002).

**Table 2:** Demographic and clinical data of the 39 subjects participating in the study, with values in parenthesis referring to 1 standard deviation.

	Alcohol Dependency	Healthy Controls	Significance
Age	56,6 (7,287)	54,8 (6,986)	n.s
Education Years	12,3 (2,536)	13,1 (2,272)	n.s
Abstinence (days)	81,6 (47,389)		
Age of drinking onset	14,6(2,836)	15,7 (1,108)	n.s
Lifetime Drinking Days	8492 (4189,731)	1982 (1659,711)	P = 0.000
Lifetime Drinks	113342 (63116,449)	9252 (10547,102)	P = 0.000
Smoking (%)	75	21	P = 0.002
MADRS^1^	10,9 (6,685)	4,5 (3,169)	P = 0.003
AUDIT^2^		2,7 (1,661)	
DUDIT^3^		0	

*1) MADRS  =  Montgomery-Asberg Depression Rating Scale 2) AUDIT  =  Alcohol Use Disorder Identification Test 3) DUDIT  =  Drug Use Disorder Identification Test.*

MADRS score difference was statistically significant (t = 3.385; P = 0.003) indicating that AD patients were more depressed than healthy controls. Five of the AD patients failed to fill in the MADRS questionnaire because of time constraints. According to the exclusion criteria no one with major depression (screening with MINI interview) was included in the study, but some of the AD patients (n = 6) suffered from mild depression (MADRS 12–20).

MINI interviews of AD patients detected previous hypomanic episodes (n = 2) and previous panic attacks (n = 1). Among the healthy controls, MINI detected cases of previous hypomanic episodes (n = 2), obsessive compulsive disorder (n = 2) and panic attacks (n = 1), but no current axis 1 psychopathology. AUDIT (mean  = 2.74; Cut off limit  = 8) and DUDIT (mean  = 0; Cut off limit  = 6) excluded current alcohol and drug abuse among the healthy controls.

Knowledge of explicit social norms evaluated by responses to 15 items from the Moral Behavior Inventory did not show any significant differences between AD patients and healthy controls (t = 0.160; P = 0.874).

A mixed ANOVA on response time with Group (AD, healthy controls) as between-subject factor, and Dilemma (Non-moral, moral impersonal and moral personal) and Response type (yes, no) as within-subject factors revealed a statistically significant effect of Group (F(1, 34)  = 9.154; P = 0.05), Dilemma (F(2, 68)  = 17.871; P = 0.000) and Dilemma x Response type interaction (F(2, 68)  = 6.520; P = 0.003). Planned comparisons showed that AD patients in general were slower to respond to all types of dilemmas compared to healthy controls (Non-moral: 8703 vs 6332 ms, P = 0.000; Impersonal: 6694 vs 4626 ms, P = 0.000; Personal: 7006 vs 5664 ms, P = 0.019). Subjects in general were slower to endorse moral personal acts (i.e. accepting the utilitarian action) compared to refusing them (6900 vs 5855 ms; P = 0.0019) while there was no significant difference between ‘yes’ and ‘no’ response times for the moral impersonal dilemmas (5875 vs 5365 ms, P = 0.149).

An item-based analysis of the moral dilemmas was also performed according to McGuire and colleagues [16] where the dependent variable was the mean response time for each individual dilemma with Dilemma (impersonal, personal) and Response type (yes, no) as factors. This yielded no significant effects of Dilemma (F(1,58)  = 0.131, P = 0.719) or Dilemma x Response type (F(1,58)  = 0.111, P = 0.740), and a trend toward an effect of response type (F(1, 58)  = 3.124, P = 0.082). After excluding four poorly endorsed dilemmas (less than 5% endorsement) we redid the subject analysis which then yielded a significant effect of Dilemma (F(1, 34)  = 6.2, P = 0.018) and Group (F(1, 34)  = 7.129, P = 0.012) and effects approaching statistical significance for Response type (F(1, 34)  = 0.079) and Dilemma x Response (F(1, 34)  = 3.493, P = 0.070).

**Figure 1:**
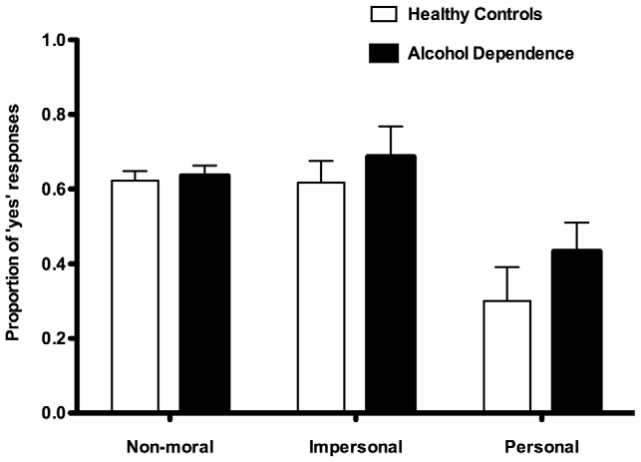
Moral judgments of three classes of dilemmas: non-moral, impersonal moral and personal moral dilemmas. The proportion of ‘yes’ responses are shown for the two groups. Alcohol dependent patients were more likely than healthy controls to respond ‘yes’, i.e. endorsing the proposed utilitarian action, when faced with moral personal dilemmas (P = 0.024). However, no such difference was found for non-moral (P = 0.377) or impersonal moral dilemmas (P = 0.161). Error bars indicate 95% confidence intervals.

Figure 1 shows the proportion of ‘yes’ responses for non-moral, moral impersonal and moral personal dilemmas. A mixed ANOVA on the proportion of ‘yes’ responses with Group (AD, healthy controls) as between-subject factor and Dilemma (Non-moral, moral impersonal and moral personal) as within-subject factor yielded a statistically significant effect of both Group (F(1, 37)  = 5.093; P = 0.030) and Dilemma (F (2, 74)  = 73.386; P = 0.000), but no significant effect of the Group x Dilemma interaction (F(2, 74)  = 2.489; P = 0.090). Planned comparison however showed that the AD patients were more likely than healthy controls to respond ‘yes’, i.e. endorsing the proposed utilitarian action, when faced with moral personal dilemmas (t = 2.350; P = 0.024), but no significant difference for neither moral impersonal (t = 1.429; P = 0.161) or non-moral (t = 0.722; P = 0.475).

**Figure 2:**
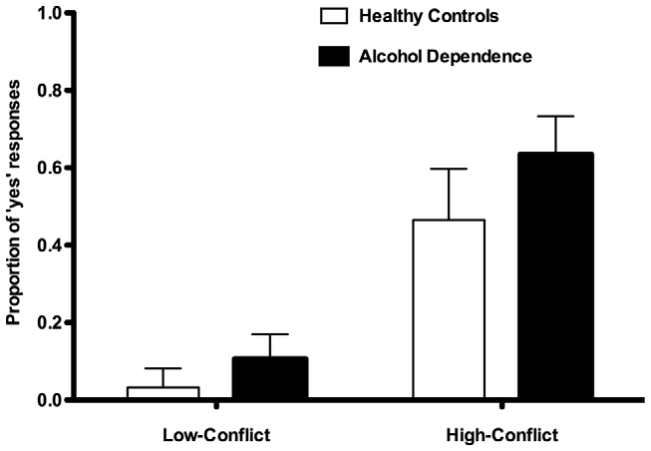
Moral judgments of the personal moral dilemmas, further subdivided into low- and high-conflict respectively. The proportion of ‘yes’ responses are shown for the two groups. Alcohol dependent patients were more likely than healthy controls to respond ‘yes’, i.e. endorsing the proposed utilitarian action, when faced with the high-conflict dilemmas (P = 0.036), while the difference was less pronounced for the low-conflict dilemmas (P = 0.063). Error bars indicate 95% confidence intervals.

**Figure 3:**
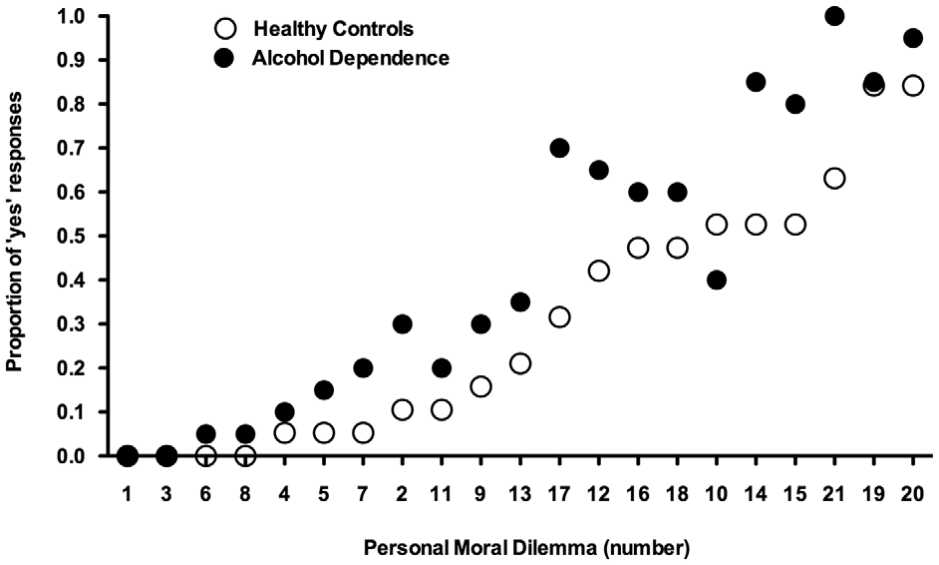
Moral judgment on each moral personal dilemma. The proportion of ‘yes’ responses are shown for the two groups for each of the 21 moral personal dilemmas. The dilemma numbers are directly adapted from Koenigs et al. (2007) and sorted according to increasing proportion of ‘yes’ responses, i.e. endorsing the proposed utilitarian action, by the healthy controls. Dilemmas labelled 1–8 and 9–21 are low-conflict type and high-conflict type respectively. Alcohol dependent patients responded equally or more utilitarian than healthy controls for all personal moral dilemmas except one, and the difference in response was more pronounced for the high-conflict dilemmas (P = 0.036) compared to low-conflict dilemmas (P = 0.063).

There was a statistically significant difference in response time on low-conflict vs high-conflict personal moral dilemmas for both AD (5680 vs 7473 ms; t = −5.387; P = 0.000) and healthy controls (4199 vs 5962 ms; t = −3.706; P = 0.02). Compared to healthy controls, AD patients gave more ‘yes’ responses when faced with the high-conflict personal dilemmas (t = 2.173; P = 0.036) while this difference exhibited a trend toward statistical significance for the low-conflict personal dilemmas (t = 1.919; P = 0.063). Figure 2 shows the proportion of ‘yes’ responses, i.e. endorsements of the proposed utilitarian action, for the moral personal dilemmas further subdivided into low- and high-conflict. AD patients responded equally or more utilitarian than healthy controls for all personal moral dilemmas except one (See figure 3).

Because of the higher proportion of mildly depressive subjects (MADRS >12) in the AD group, we performed a post hoc analysis to determine the influence of MADRS score on utilitarian moral judgment. A mixed ANOVA on proportion of ‘yes’ responses with Group (Mild Depression, No Depression) as between-subject factor and Dilemma (Non-moral, moral impersonal and moral personal) as within-subject factor. This revealed a significant effect of Dilemma (F(2, 64)  = 34.90; P = 0.000), a trend toward an effect of Group (F(1, 32)  = 3.28; P = 0.08 ) and no significant Group x Dilemma interaction ( F(2, 64)  = 1.356; P = 0.265). In a post-hoc t-test on proportion of ‘yes’ responses, study subjects with mild depression (MADRS >12) were compared to study subjects without mild depression (MADRS <12). There was no difference regarding responses to impersonal moral judgments (t = 0.707; P = 0.485), but subjects with mild depression exhibited a trend toward responding more utilitarian compared to subjects without depression (t = 1.90; P = 0.066)

## Discussion

The results of this case-control study of AD patients and healthy controls confirmed the hypothesis that AD patients generate increased utilitarian moral judgments when faced with moral personal dilemmas, compared to healthy controls. The subjects responded to a battery of dilemmas divided into non-moral, moral impersonal and moral personal dilemmas (further subdivided into low- and high-conflict), and 15 items evaluating knowledge of explicit social and moral norms. Since AD patients exhibit impairments in functions mediated by the PFC [25], as well as similar decision making as VMPC lesion patients [34], AD patients were hypothesized to generate increased utilitarian moral judgment while having intact knowledge of explicit social and moral norms, similar to VMPC lesion patients [18], [19].

The AD patients were slower than healthy controls to respond to the dilemmas in general, which could be explained by slower reading pace and comprehension, or unfamiliarity with computer administered tasks. Also, since the subjects were encouraged to ask questions when they did not understand something in the presented dilemma, the response time data is not optimal and should thus be interpreted with caution. The present response time data replicate findings from previous research employing the same moral dilemma battery, as subjects in general were slower to endorse moral personal acts compared to refusing them, while there was no significant difference between ‘yes’ and ‘no’ response times for the moral impersonal dilemmas [7], [8], [14]. Also, subjects within each group responded faster to low-conflict compared to high-conflict personal moral dilemmas, which replicates the previous findings and confirms this subdivision of moral personal dilemmas [15]. However, we also performed an item analysis in which the response time was analysed across each of the different dilemmas instead of subjects, according to previous research criticising the original distinction between impersonal/personal moral dilemmas [16]. This analysis yielded no significant effects, which suggests that the observed differences in response times in the subject analysis were driven by a small subset of dilemmas, which questions the postulated distinction between personal and impersonal moral dilemmas. Thus, the present response time results should be viewed in light of the fact there are methodological problems related to the moral dilemma battery and the subdivisions of moral dilemmas (for further discussion, see [15], [16]).

No difference was found between groups in responses to non-moral and moral impersonal dilemmas, nor regarding knowledge of explicit social and moral norms. This implicates that the difference in moral judgment does not depend on a more general decision making deficit, nor is it explained by failure to understand social and moral norms. However, AD patients were more likely than healthy controls to respond ‘yes’, i.e. endorsing the proposed utilitarian act, when faced with personal moral dilemmas. This difference was greatest for the subgroup of personal moral dilemmas classified as “high-conflict”, such as the “plane crash”-dilemma described above, where an emotionally aversive act is required to maximize aggregate welfare. Figure 3 illustrates responses to all individual personal dilemmas for each group, ordered by increasing proportion of ‘yes’ responses in the healthy control group. Our results confirm the validity of the subdivision of personal dilemmas made by Koenigs et al. [19], regarding low-conflict dilemmas (labelled 1–8) and high-conflict dilemmas (9–21), as the observed difference between groups emerges more clearly in the high-conflict dilemmas. According to the ‘dual-process theory of moral judgment’ [8], high-conflict moral dilemmas induce a conflict between cognition and emotion, and a utilitarian moral judgment depends on cognitive processes overriding the emotional response. However, in patients who suffer from emotional dysfunction caused by VMPC dysfunction, the “high-conflict” dilemmas do not induce the same degree of conflict between cognition and emotion, and thus these patients are more likely to generate the utilitarian moral judgment [8], [18], [19].

The somatic marker theory stipulates that the neural substrates responsible for homeostasis, emotion and feelings fundamentally determine our decision-making in general [6]. Verdejo-Garcia and Bechara [34] has proposed a ‘somatic marker theory of addiction’, based on the growing evidence of similarities in decision making between VMPC lesion and substance abuse patients, namely their tendency to choose an immediate reward while disregarding the long-term negative consequences. According to this view, there is a link between the emotional dysfunction and altered decision making in substance abusers. The present study implicates that this is also true for complex moral decision making, since AD patients exhibit a similar pattern of moral judgment as VMPC lesion patients [18], [19].

Conclusions regarding underlying neuronal processes determining the increased utilitarian moral judgment in AD patients cannot be made based on this data set. However, according to the ‘frontal lobe hypothesis’, the neuropsychological profile of AD patients (e.g. increased impulsivity, risky decision making, impaired emotional facial perception) is caused by specific neurotoxic effects of alcohol on the PFC [25]. If this hypothesis is true, the present results would indicate that the neurotoxic effects of alcohol on the VMPC causes emotional dysfunction, which results in increased utilitarian moral judgment to emotionally salient moral dilemmas. However, the ‘frontal lobe hypothesis’ does not differentiate between different functional areas of the PFC. AD patients also show impairments of e.g. working memory, indicating dysfunction of the dorsolateral PFC [27], which is one of the ‘cognitive’ neural areas associated with utilitarian moral judgment according to the ‘dual process theory of moral judgment’ [7], [8]. One possible explanation of the present results could be that even though alcohol causes wide spread damage to the PFC, including both ‘cognitive’ and ‘emotional’ areas, the aggregate effect results in a relatively greater impairment of ‘emotional’ function when faced with moral dilemmas. Thus, perhaps the cognitive ability to compare 1 versus 5 lives is more preserved in AD patients, compared to the ability to generate an emotional response when faced with an emotionally salient moral dilemma.

Early studies of moral judgment in AD patients found no difference between AD patients and healthy controls, evaluated by the Kohlberg scale of moral maturity [41]. However, according to Kohlberg the trademark of high level of moral maturity is rational moral reasoning from explicit universal principles concerning welfare or human rights [4]. In the present study, moral reasoning in AD patients was by no way impaired according to the standards of Kohlberg. Rather, AD patients exhibited a tendency toward a more rational utilitarian way of moral reasoning, based on the universal principle of maximizing aggregate welfare, which at least according to utilitarian moral philosophers would be a superior way of moral reasoning. This elucidates a novel aspect of the neuropsychological profile of AD patients, namely a tendency towards a more rational, but less emotional, way of moral reasoning. This distinction is not captured by the Kohlberg scale of moral maturity, which explains why earlier studies did not find any difference in moral judgment between AD patients and healthy controls. However, to what degree increased utilitarian moral judgment is specific to AD compared to other types of addiction, and whether this utilitarian bias translates into addiction related behaviour, e.g. tendency to relapse despite negative emotional consequences, remains to be answered.

This study had several limitations. Firstly, the sample size is limited and the data should therefore be interpreted with caution until replicated. Secondly, even though the groups were adequately matched regarding sex, age and education years, there was still a mismatch since AD patients had higher MADRS scores and a higher proportion of smokers. Further, our post hoc analysis showed that patients with mild depression (MADRS >12) showed a trend towards more utilitarian responses compared to the subjects without mild depression (MADRS <12). This suggests that depressive symptoms might constitute a confounding factor in the present study. However, it should be noted that none of the AD patients in our study fulfilled the DSM-IV criteria for major depression. Furthermore, it is well known that long term drug intake induces persistent neuroadaptations in the brain [22], resulting in a state of increased negative affect mediated by down regulation of reward pathways and up regulation of brain stress circuits [42]. Thus, negative affect and depressed mood in the absence of alcohol constitute an intrinsic part of the disease of AD. Future studies are needed to further investigate the role of depressive symptoms in moral judgment, by focusing for instance on patients with mood disorders without co-morbid substance abuse.

Finally, it is important to note the limitations of case-control studies regarding the question of causality. Whether life long alcohol intake causes an increased tendency towards utilitarian moral judgment, or if individuals with a predisposition for utilitarian moral judgment are more likely to develop AD, remains unanswered. Finally, the data in this study is purely behavioural. Thus, the discussion above regarding emotional dysfunction related to PFC dysfunction should be viewed as speculative until functional imaging data confirms the hypothesis. Since moral reasoning is a complex function involving several brain areas besides the VMPC, such as medial frontal gyrus, posterior cingulate, superior temporal sulcus region, the temporal pole, amygdala and dorsolateral PFC (for review see [43]), altered moral reasoning could hypothetically result from abnormal function in any of these other brain structures. For instance, it is well established that addiction patients have an altered function in several brain regions besides the PFC, e.g. the dopaminergic mesolimbic system, amygdala and hippocampus (for review see [22]). It is thus possible that the present finding of increased utilitarian moral judgment in AD patients is caused by dysregulation of these subcortical brain structures, rather than prefrontal regions.

In conclusion, our results indicate that AD patients generate increased utilitarian moral judgment when faced with emotionally salient moral personal dilemmas. The importance of these findings is two-fold. Firstly, they represent new evidence in support of the ‘frontal lobe hypothesis’ [25] of the neuropsychological profile in AD patients and the ‘somatic marker theory of addiction’ [34], as well as the ‘dual-process theory of moral judgment’ [8]. Secondly, they increase our understanding of the neuropsychological profile of AD patients. When faced with moral personal dilemmas, this patient group has a propensity to generate utilitarian moral judgment. Further research in the intersection of psychiatry and moral psychology could improve our understanding of complex decision making and inter-personal behaviour in psychiatric clinical populations.
